# Assessment of the Impact of Humic Acids on Intestinal Microbiota, Gut Integrity, Ileum Morphometry, and Cellular Immunity of Turkey Poults Fed an Aflatoxin B_1_-Contaminated Diet

**DOI:** 10.3390/toxins16030122

**Published:** 2024-02-29

**Authors:** Jesús A. Maguey-González, Jing Liu, Guolong Zhang, Juan D. Latorre, Juan O. Hernández-Ramírez, María de Jesús Nava-Ramírez, Roberto Senas-Cuesta, Sergio Gómez-Rosales, María de Lourdes Ángeles, Andressa Stein, Bruno Solís-Cruz, Daniel Hernández-Patlán, Rubén Merino-Guzmán, Xochitl Hernandez-Velasco, Inkar Castellanos-Huerta, Santiago Uribe-Diaz, Alma Vázquez-Durán, Abraham Méndez-Albores, Victor M. Petrone-Garcia, Guillermo Tellez Jr., Billy M. Hargis, Guillermo Téllez-Isaías

**Affiliations:** 1 Department of Poultry Science, University of Arkansas, Fayetteville, AR 72701, USA; jl11@uark.edu (J.D.L.); rsenascu@uark.edu (R.S.-C.); andressastein.s@gmail.com (A.S.); icastell@uark.edu (I.C.-H.); su004@uark.edu (S.U.-D.); bhargis@uark.edu (B.M.H.); gtellez@uark.edu (G.T.-I.); 2 Department of Animal and Food Sciences, Oklahoma State University, Stillwater, OK 74078, USA; glenn.zhang@okstate.edu; 3 Unidad de Investigación Multidisciplinaria L14 (Alimentos, Micotoxinas, y Micotoxicosis), Facultad de Estudios Superiores (FES) Cuautitlán, UNAM, Cuautitlán Izcalli 54740, Mexico; mvzjohr@cuautitlan.unam.mx (J.O.H.-R.); mari_551293@comunidad.unam.mx (M.d.J.N.-R.); almavazquez@comunidad.unam.mx (A.V.-D.); albores@unam.mx (A.M.-A.); 4 Centro Nacional de Investigación Disciplinaria en Fisiología y Mejoramiento Animal (CENID-INIFAP), Km1 Carretera a Colon Ajuchitlán, Querétaro 76280, Mexico; gomez.sergio@inifap.gob.mx (S.G.-R.); angeles.lourdes@inifap.gob.mx (M.d.L.Á.); 5 Laboratorio 5: LEDEFAR, Unidad de Investigación Multidisciplinaria, Facultad de Estudios Superiores (FES) Cuautitlán, UNAM, Cuautitlán Izcalli 54740, Mexico; bruno_sc@comunidad.unam.mx (B.S.-C.); danielpatlan@comunidad.unam.mx (D.H.-P.); 6 División de Ingeniería en Nanotecnología, Universidad Politécnica del Valle de México, Tultitlan 54910, Mexico; 7 Departamento de Medicina y Zootecnia de Aves, Facultad de Medicina Veterinaria y Zootecnia, UNAM, Ciudad de México 04510, Mexico; onirem@unam.mx (R.M.-G.); xochitlh@fmvz.unam.mx (X.H.-V.); 8 Facultad de Estudios Superiores (FES) Cuautitlán, UNAM, Cuautitlán Izcalli 54740, Mexico; 9 Department of Developmental Biology, Roslin Institute, University of Edinburgh, Edinburgh EH25 9RG, UK; memotellez98@gmail.com

**Keywords:** turkey poults, aflatoxin B_1_, humic acids, intestinal microbiota, gut integrity, morphometric studies

## Abstract

A recent study published data on the growth performance, relative weights of the organs of the gastrointestinal tract, liver histology, serum biochemistry, and hematological parameters for turkey poults fed an experimental diet contaminated with aflatoxin B_1_ (AFB_1_) and humic acids (HA) extracted from vermicompost. The negative effects of AFB_1_ (250 ng AFB_1_/g of feed) were significantly reduced by HA supplementation (0.25% *w*/*w*), suggesting that HA might be utilized to ameliorate the negative impact of AFB_1_ from contaminated diets. The present study shows the results of the remaining variables, as an extension of a previously published work which aimed to evaluate the impact of HA on the intestinal microbiota, gut integrity, ileum morphometry, and cellular immunity of turkey poults fed an AFB_1_-contaminated diet. For this objective, five equal groups of 1-day-old female Nicholas-700 turkey poults were randomly assigned to the following treatments: negative control (basal diet), positive control (basal diet + 250 ng AFB_1_/g), HA (basal diet + 0.25% HA), HA + AFB_1_ (basal diet + 0.25% HA + 250 ng AFB_1_/g), and Zeolite (basal diet + 0.25% zeolite + 250 ng AFB_1_/g). In the experiment, seven replicates of ten poults each were used per treatment (*n* = 70). In general, HA supplementation with or without the presence of AFB_1_ showed a significant increase (*p* < 0.05) in the number of beneficial butyric acid producers, ileum villi height, and ileum total area, and a significant reduction in serum levels of fluorescein isothiocyanate–dextran (FITC-d), a marker of intestinal integrity. In contrast, poults fed with AFB_1_ showed a significant increase in Proteobacteria and lower numbers of beneficial bacteria, clearly suggesting gut dysbacteriosis. Moreover, poults supplemented with AFB_1_ displayed the lowest morphometric parameters and the highest intestinal permeability. Furthermore, poults in the negative and positive control treatments had the lowest cutaneous basophil hypersensitivity response. These findings suggest that HA supplementation enhanced intestinal integrity (shape and permeability), cellular immune response, and healthier gut microbiota composition, even in the presence of dietary exposure to AFB_1_. These results complement those of the previously published study, suggesting that HA may be a viable dietary intervention to improve gut health and immunity in turkey poults during aflatoxicosis.

## 1. Introduction

Aflatoxin B_1_ (AFB_1_), a potent mycotoxin primarily produced by *Aspergillus flavus, A. parasiticus* and *A. nomius*, poses a significant threat to poultry health and productivity. Its presence in poultry feed can lead to various detrimental effects, including reduced growth performance, impaired nutrient absorption, compromised intestinal integrity, and suppressed immune function [1]. In turkey poults, the negative consequences of AFB_1_ exposure are particularly pronounced, leading to an increased susceptibility to bacterial and viral infections and mortality [2].

Several researchers have demonstrated the detrimental impact of AFB_1_ on turkey poults through productive parameters since there is a marked reduction in the body weight gained and feed efficiency [3]. AFB_1_ disrupts nutrient absorption and utilization, decreasing feed intake and conversion efficiency and ultimately impacting growth and development. Moreover, impaired intestinal integrity by damage in the intestinal epithelium compromises the barrier function and facilitates the entry of pathogens and their toxins [4]. This can lead to increased intestinal permeability and inflammatory responses [5], as well as suppressed cellular and humoral immune responses, rendering turkey poults more susceptible to opportunistic infections and disease outbreaks [6].

Several approaches to mycotoxin-mitigation strategies have been conducted, encompassing physical methods (irradiation), chemical treatments (oxidizing agents), biological methods (microorganisms, enzymes), and nutritional regulation approaches. Despite their effectiveness, limitations such as inefficiency, high costs, and scalability hinder extensive adoption. This context leads to a requirement for the development of novel and environmentally friendly technologies to address this growing concern within the framework of environmental protection and food safety [7,8,9,10,11].

Recent in vitro and in vivo studies have established the efficacy of humic acids (HA) in mitigating the detrimental effects of AFB_1_ in turkey poults. These benefits include an improved performance and a significant reduction in aflatoxicosis severity [12,13]. Given the promising findings regarding the benefits of HA in mitigating AFB_1_ toxicity in poultry, the present study aimed to assess the impact of dietary HA supplementation on various aspects of turkey poult health (intestinal microbiota, gut integrity, ileum morphometry, and cellular immunity) when fed an AFB_1_-contaminated diet.

Furthermore, despite the promising findings of recent studies, the intricate mechanisms by which HA modulates the intestinal microbiota and counteracts AFB_1_ still need to be explored. Understanding these mechanisms is crucial for optimizing HA supplementation strategies and maximizing their beneficial impact on turkey poults’ health. This study delves deeper into the unexplored territory of the gut microbiota. We aim to comprehensively assess the impact of HA supplementation on the microbial composition of AFB_1_-challenged turkey poults. Through advanced sequencing and bioinformatic analyses, we characterized the shifts in gut microbiota diversity and abundance to evaluate the insights into which bacterial populations are most affected by AFB_1_ exposure and how HA influences their composition and identify key microbial players to pinpoint specific bacterial taxa that potentially contribute to AFB_1_ detoxification or play a role in mitigating its adverse effects. Additionally, we sought to elucidate the potential mechanisms that may correlate changes in the gut microbiota with alterations in gut integrity, ileum morphometry, and immune responses; these might provide a better understanding of the mechanisms by which HA modulate the gut environment and protect turkey poults during aflatoxicosis. By investigating these key parameters, this study aims to provide a comprehensive understanding of the potential of HA as a dietary supplement for mitigating the harmful effects of AFB_1_ in turkey poults. This knowledge can contribute to the development of safe and cost-effective strategies for safeguarding turkey health and promoting sustainable poultry production practices.

## 2. Results

The results of the relative abundances (%) of the cecal bacterial Phyla and their families among different treatments are summarized in Table 1. At the Phyla level, a significant increase in Proteobacteria was observed in poults fed with AFB_1_ (PC) and poults that received ZEO + AFB_1_ (*p* < 0.05). This increase was associated with a significant reduction in Firmicutes. In contrast, poults that received HA or HA + AFB_1_ had the opposite effect: a significant increase in Firmicutes and a significant reduction in Proteobacteria. Similar effects were observed at the family level. PC treatment showed a significant increase in Enterobacteriaceae and a significant reduction in Lachnospiraceae and Peptostreptococcaceae compared to HA or HA + AFB_1_-treated poults (Table 1).

Moreover, Table 2 shows the results of the relative abundances (%) of the cecal bacterial genera and amplicon sequence variants (ASV) among different treatments. A similar trend was observed at the genera and ASV levels. PC and ZEO + AFB_1_ treatments showed a significant increase in *Escherichia/Shigella* compared to poults that received HA and HA + AFB_1_. Meanwhile, PC treatment was shown to lead to a significant reduction in the presence of *Anaerosipes* in comparison to the rest of the treatments (Table 2).

**Figure 1:**
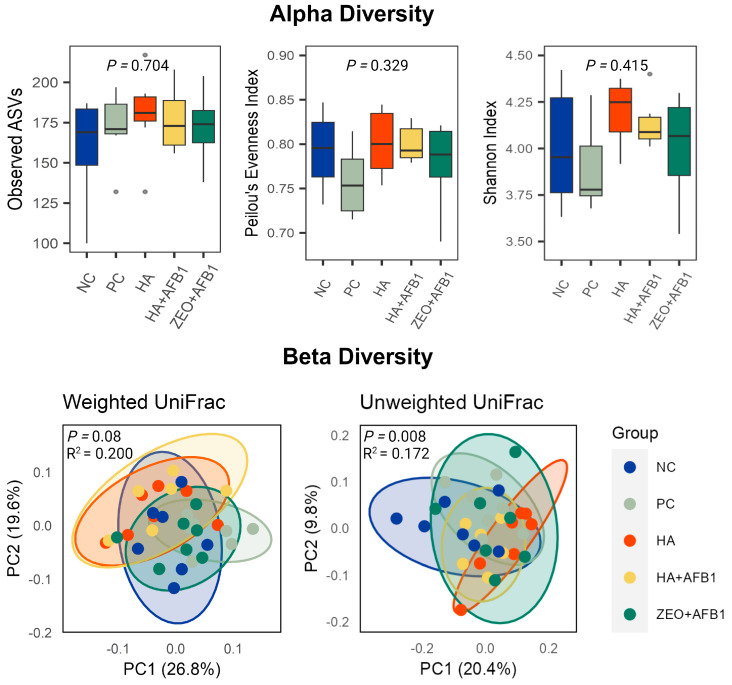
Alpha and beta diversities of the cecal microbiota among different experimental treatments. The cecal contents (*n* = 7/treatment) were subjected to 16S rRNA gene sequencing. Observed ASV, Pielou’s Evenness, and Shannon Index were calculated to measure the α-diversity of the cecal microbiota; Kruskal-Wallis test was used for statistical significance determination. The β-diversity-weighted UniFrac and unweighted UniFrac distances were used to generate the principal coordinates analysis (PCoA) plots. Permutational multivariate analysis of variance (PERMANOVA) was used for statistical significance determination. NC—negative control; PC—positive control (basal diet + 250 ng AFB_1_/g); HA—humic acids (basal diet + 0.25% HA); HA + AFB_1_(basal diet + 0.25% HA + 250 ng AFB_1_/g); ZEO—Zeolite (basal diet + 0.25% ZEO + 250 ng AFB_1_/g).

Moreover, Figure 1 shows the results of the alpha and beta diversities of the cecal microbiota among different treatments. For α-diversity, no significant differences among treatments were observed. The PC and ZEO + AFB_1_ treatments had a numerical trend of decreased Peilou’s Evenness and Shannon index (measures the overall alpha diversity of the bacterial community) compared to the HA + AFB_1_ treatment, indicating that the challenge of AFB_1_ could potentially cause the proliferation of certain pathogenic bacteria, leading to a change in the bacteria community. However, HA supplementation could restore this decreased evenness of bacteria to healthy levels (Figure 1).

Based on weighted UniFrac distance, there was a significant divergence in β-diversity between the NC and PC treatments, indicating the influence of AFB_1_ on the microbial community composition. However, no significant differences were observed between NC and HA or between NC and ZEO + AFB_1_. Still, it is suggested that the supplementation of HA and Zeolite could restore the intestinal microbiota (β-diversity) to healthy levels (Figure 1).

**Figure 2:**
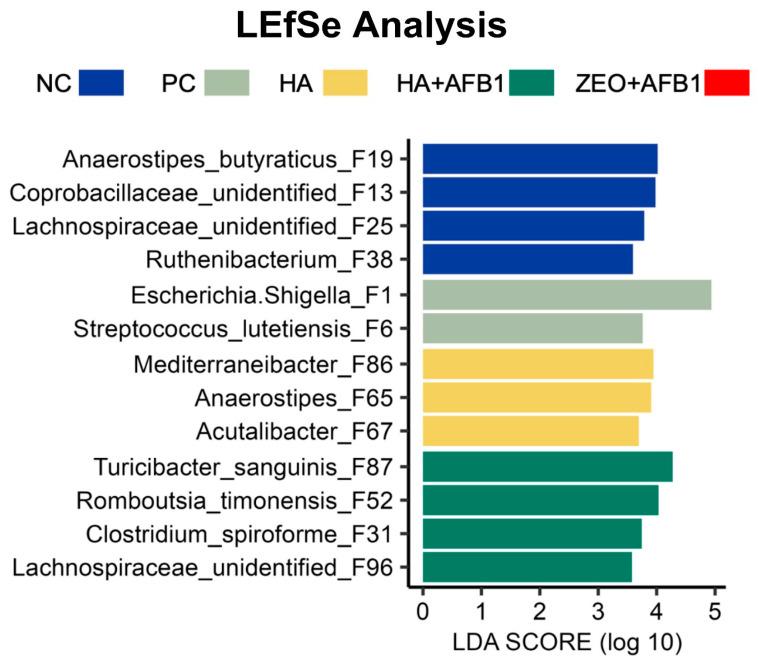
Differential enrichment of bacterial ASV between different experimental treatments (*n* = 7/treatment) was determined using linear discriminant analysis (LDA) effect size (LEfSe), with the all-against-all multiclass analysis, *p*  <  0.05, and a logarithmic LDA threshold of 3.0. NC—negative control; PC—positive control (basal diet + 250 ng AFB_1_/g); HA—humic acids (basal diet + 0.25% HA); HA + AFB_1_(basal diet + 0.25% HA + 250 ng AFB_1_/g); ZEO—Zeolite (basal diet + 0.25% ZEO + 250 ng AFB_1_/g).

The results of differential enrichment levels of bacterial ASVs between the different treatments were determined using LEfSe, employing an all-against-all multiclass analysis approach (Figure 2). The data from all experimental treatments for LEfSe analysis were used to identify several bacterial species that were enriched in the NC, PC, HA, and HA + AFB_1_ treatments but not in the ZEO + AFB_1_ treatment. For example, *Anaerostipes butyraticus* was found to be enriched in the NC treatment compared to the rest of the treatments based on LEfSe analysis. The absence of any species from the ZEO + AFB_1_ treatment in the LEfSe analysis indicates that no bacterial species in this treatment were significantly more abundant than in the other treatments. Therefore, the absence of the ZEO + AFB_1_ indicates that no bacterial species were found to be enriched in the ZEO + AFB_1_ treatment in comparison to the other four experimental treatments (Figure 2).

**Figure 3:**
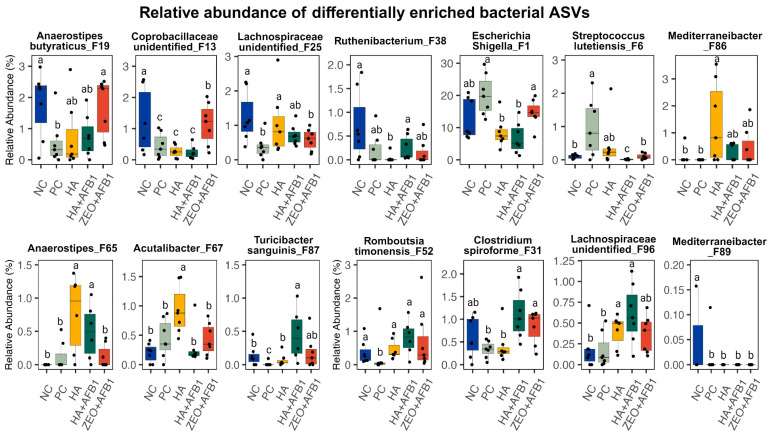
Relative abundances of differentially enriched bacterial ASV (*n* = 7/treatment). Significance was calculated using Kruskal–Wallis test. ^a,b,c^ Indicates significant differences between the treatments (*p* < 0.05). NC—negative control; PC—positive control (basal diet + 250 ng AFB_1_/g); HA—humic acids (basal diet + 0.25% HA); HA + AFB_1_ (basal diet + 0.25% HA + 250 ng AFB_1_/g); ZEO—Zeolite (basal diet + 0.25% ZEO + 250 ng AFB_1_/g).

Figure 3 shows the results of the relative abundance of differentially enriched bacterial ASV. A significant increase in *Escherichia*, *Shigella*, and *Streptococcus lutetiensis* can be observed under the PC treatment. This was also associated with a significant reduction in beneficial bacteria compared with the HA, HA + AFB_1_, and ZEO + AFB_1_ treatments, confirming that AFB_1_ induced dysbacteriosis in poults. In contrast, poults fed with HA showed a significant increase in the numbers of beneficial bacteria such as *Mediterraneibacter*, *Anaerostipes*, and *Acutalibacter*. Interestingly, the poults of the HA + AFB_1_ treatment also showed a significant increase in the number of beneficial bacteria (butyric acid producers), such as *Turicibcter sanguinis*, *Rombusti timonensis*, *Clostridium spiroforme*, and *Lachnospiraceae* sp. (Figure 3).

The results of the effect of HA on morphometric analysis, serum levels of fluorescein isothiocyanate dextran (FITC-d), and cutaneous basophil hypersensitivity response (CBH) in turkeys consuming a diet contaminated with 250 ng of AFB_1_/g for 28 d are summarized in Table 3. Poults in the NC, HA, and HA + AFB_1_ treatments showed a significant increase in villi height followed by poults in the ZEO + AFB_1_ treatment. In contrast, the PC treatment showed the lowest villi height. Poults in the NC and HA + AFB_1_ treatments showed the highest total area, followed by poults supplemented with HA and ZEO + AFB_1_ (Table 3, Figure S1). Meanwhile, the PC treatment had the lowest total area and displayed the highest serum concentration of FITC-d compared to the rest of the experimental treatments. Moreover, poults supplemented with HA + AFB_1_ and ZEO + AFB_1_ showed a significant increase in CBH response, followed by HA and PC treatments. On the contrary, the NC treatment had the lowest CBH response. 

## 3. Discussion

This study expands previous research by looking into the varied effects of HA on turkey poults fed an AFB_1_-contaminated diet. The observed benefits of HA on intestinal microbiota, gut integrity, ileal morphometry, and cellular immunity significantly strengthen the argument for their use as a practical and effective intervention against AFB_1_ toxicity. These positive effects align with previous publications [12,13]. The improved villus height and width, indicative of enhanced nutrient absorption, corroborate the findings from Taklimi et al. and López-García et al. [14,15]. Similarly, the reduced intestinal permeability aligns with the findings presented by Maguey-Gonzalez et al. [16], suggesting HA’s ability to strengthen the gut barrier and prevent AFB_1_ absorption.

This investigation delves further into the effects of HA on the gastrointestinal microbiota and cellular immunity. The observed shift towards beneficial Firmicutes and reduced Proteobacteria, both known for their influence on gut health and immune function, is novel and promising [17]. Additionally, the enhanced CBH in HA-fed poults suggests a boost in cellular immunity, potentially contributing to improved resistance against AFB_1_-induced inflammation.

The findings of this study also solidify the potential of HA as a valuable tool in mitigating AFB_1_’s detrimental effects on turkey poults. By improving gut integrity, promoting beneficial gut bacteria, improving intestinal morphometry, and enhancing cellular immunity, HA can potentially reduce performance losses, improve feed conversion ratio, and increase body weight gain, according to a previously published study [18]. Enhanced gut health can also protect poults from AFB_1_-induced intestinal damage, inflammation, and secondary bacterial or viral infections.

The potential of HA to increase Firmicutes in poults, both with and without AFB_1_ exposure, is an intriguing area of research with promising implications for poultry health and mycotoxin control. Some of the potential mechanisms for increased Firmicutes with HA may include the following: (A) A prebiotic effect—HA, particularly fulvic acid, contain complex organic molecules with prebiotic properties [18]. These molecules can stimulate the growth of beneficial bacteria, including Firmicutes, by providing them with readily available energy sources and enhancing their metabolic activity [19]. HA may also act as natural surfactants, increasing the permeability of cell membranes in bacteria, due to their amphiphilic character, which enhances the absorption of nitrogen and other micronutrients [20]. (B) Binding and detoxification of AFB_1_—HA have a high affinity for AFB_1_, binding to it mainly via hydrogen bonding and reducing its bioavailability in the intestine [12]. This effect reduces the toxicity of mycotoxin, protecting Firmicutes from its detrimental effects and potentially allowing them to thrive. (C) Anti-inflammatory and immune-modulatory properties [21]—HA can suppress the inflammatory response triggered by AFB_1_ and stimulate the immune system, generating a more favorable environment for Firmicutes to flourish. (D) Improvement of gut barrier function—HA can improve gut barrier integrity by enhancing the production of intestinal epithelial cells and tight junctions [22]. Results showing increased gut viscosity and gene expression of mucin 2 (MUC-2) in the cecum of chickens consuming HA [23] also suggest greater mucin production in poultry. This strengthened barrier reduces the entry of AFB_1_ and other harmful/toxic substances. (E) Alteration of gut microbiota composition—HA might directly affect the composition of the gut microbiota, potentially favoring *Firmicutes* over other bacterial groups like Proteobacteria [24]. For instance, in weaned, HA-fed Holstein calves, the relative abundance of Firmicutes increased, while a decrease in Bacteroidetes was reported. This effect could be due to the selective prebiotic effect or other mechanisms involving competition for resources and niche differentiation [25].

On the other hand, AFB_1_ is a potent mycotoxin commonly found in poultry feed, which can significantly disrupt the delicate balance of gut microbiota [26]. This disruption often leads to an overgrowth of Proteobacteria, a group of typically Gram-negative bacteria that are not typically dominant in a healthy chicken gut. Moreover, AFB_1_ exposure triggers a stress response in chickens, leading to immune suppression and inflammation [27]. This weakened immune system becomes less efficient in controlling the growth of opportunistic pathogens like Proteobacteria, which can readily exploit the situation and proliferate, causing dysbacteriosis and intestinal inflammation; these are commonly associated with increased permeability and chronic systemic inflammation [28]. In the present study, poults fed with AFB_1_ showed a significant increase in Proteobacteria and a significant reduction in Firmicutes. This selective pressure can lead to a decrease in Firmicutes and a relative increase in Proteobacteria, which are more tolerant to certain mycotoxins or their combinations. Interestingly, poults fed with HA showed the opposite results, even in the presence of the mycotoxin.

It is well known that some Proteobacteria species possess enzymes that can metabolize AFB_1_ and its breakdown products [29]. This ability allows them to thrive in the presence of mycotoxins, while other bacterial groups struggle. Furthermore, AFB_1_ exposure can alter pH and nutrient availability in the gut mucosa, generating a more favorable environment for certain Proteobacteria species to thrive [30].

On the other hand, LEfSe pinpoints differentially enriched bacteria across groups, based on the relative abundance of individual bacterial species [31]. An enrichment in a particular group suggests a higher abundance of a species in that group compared to others. This method helps infer an increase in specific bacterial species in each group, relative to others. In multi-group comparisons, a highlighted species denotes its predominant relative abundance [32]. LEfSe analysis revealed that poults that received the supplementation of HA and a diet contaminated with AFB_1_ had a relative abundance of differentially enriched bacterial ASV of Lachnospiraceae, *Turicibacter*, *Romboutsia*, and *Clostridium*. Lachnospiraceae is a large family of bacteria within the Firmicutes phylum, commonly found in the guts of humans and animals, and even in the environment [33]. They are part of the Class Clostridia and Order Eubacteriales. Lachnospiraceae are known for their ability to produce short-chain fatty acids (SCFAs) like butyrate, acetate, and propionate [34,35]. These SCFAs have various benefits for poultry, as they provide a readily available energy source for gut epithelial cells [36], promoting intestinal health and barrier function [37], promoting immune modulation [38], reducing inflammation, protecting against pathogens [39], stimulating the absorption of certain micronutrients like calcium and magnesium [40], promoting fiber degradation [41], and the promoting production of antimicrobial compounds that inhibit the growth of harmful bacteria, potentially reducing the risk of infections [42].

In other animal species supplemented with HA, increased production of SCFAs has been also reported. For instance, in HA-fed dairy cows, increased total volatile fatty acids and increased proportions of acetate and propionate contents in the rumen were observed [43]. In humic substance (HS)-fed goats, increased concentrations of ruminal acetate and propionate were reported [44]. Furthermore, in rabbits consuming HS, an increase in the cecal concentration of propionic and butyric acids was observed [45]. These results reinforce the suggestion that HA causes a shift in the digestive microbiota by stimulating the growth of probiotic-type bacteria and modifying the microbial fermentation in non-ruminant and ruminant animals, leading to the formation of a greater amount of SCFAs [19,46].

The importance of *Turicibacter sanguinis* in poultry is not yet fully understood, as this relatively recently discovered bacterial species is still being actively researched [47]. However, some potential roles and impacts are starting to emerge, due to its ability to degrade complex dietary fibers, such as cellulose and hemicellulose [48]. This makes the nutrients locked within these fibers accessible to the poultry, potentially improving their overall nutrient utilization and feed efficiency. During fiber degradation, *T. sanguinis* produces beneficial SCFAs like acetate, propionate, and butyrate.

Unfortunately, research on the specific importance of *Romboutsia timonensis* in poultry is very limited. This bacterium was identified in 2016, and its role in poultry gut microbiota is still largely unknown [49]. Nevertheless, we can speculate on its potential significance based on what we know about other members of the *Lachnospiraceae* family to which *R. timonensis* belongs [24,50]. Interestingly, poults that received AFB_1_ showed a significant reduction in these important butyric acid producers.

Recent studies have shown that HA can significantly increase the size and absorption area of the villi in jejunum and ileum of rats fed an AFB_1_-contaminated diet [22], which clearly agrees with the results of the present study. Several mechanisms might be responsible for the observed increase in ileum villi size and absorption area in HA-fed poults. Previous in vitro studies demonstrated that HA have a strong affinity for AFB_1_ due to their complex molecular structures. This binding reduces the amount of free AFB_1_ molecules available for absorption, preventing their harmful effects on gut health and nutrient absorption [12]. Moreover, in another recent study, we confirmed the anti-inflammatory effects of HA on poults that received AFB_1_-contaminated feed [13]; those results are also associated with the immunomodulatory properties of HA on the CBH response. Hence, the anti-inflammatory effect of HA may reduce the intestinal inflammation caused by AFB_1_, promoting tissue repair and regeneration, which can lead to increased villi size and function.

Furthermore, the microbiota results of the present study suggest that HA can act as prebiotics, stimulating the growth of beneficial bacteria, particularly Firmicutes. These bacteria contribute to better intestinal health by producing SCFAs that promote gut barrier function, villi growth, and nutrient absorption. Larger villi size and a larger absorption area in the ileum translates to several advantages for turkey poults, such as improved nutrient absorption of essential nutrients from the feed, leading to better growth and performance.

The observed effects of HA on the morphometric analysis of the ileum mucosa are even more pronounced when compared to the positive control poults, which received the AFB_1_-contaminated feed without HA supplementation. This comparison highlights the effectiveness of HA in mitigating the negative impacts of AFB_1_ on gut health and function.

FITC-d is a large, non-digestible molecule that is commonly used as a biomarker to assess intestinal permeability. Normally, the gut barrier restricts the passage of large molecules like FITC-d into the bloodstream [51]. However, when intestinal damage occurs, FITC-d can leak into the circulation, leading to elevated serum levels. Therefore, the observed reduction in FITC-d in HA-fed poults suggests an improved intestinal barrier function and a reduced permeability. These results confirm those of previous studies that have shown a significant reduction in serum FITC-d in chickens supplemented with HA [16]. Multiple pathways could explain how HA contribute to the decrease in FITC-d in AFB_1_-challenged poults. For instance, HA have a strong affinity for AFB_1_, forming complexes that prevent its absorption in the gut [12]. This reduces the toxic effects of AFB_1_ on the intestinal epithelium, potentially minimizing damage and maintaining gut barrier integrity. Moreover, HA possess antioxidant activity, scavenging free radicals generated by the AFB_1_ metabolism [52]. This can protect the gut cells from oxidative stress and subsequent damage, preventing permeability loss. Furthermore, HA can act as prebiotics, stimulating the growth of beneficial bacteria [53]. These bacteria contribute to gut health by producing SCFAs that strengthen the intestinal barrier and reduce inflammation [54,55]. Finally, HA can modulate the immune system, reducing inflammation and promoting tissue repair [56]. This could help repair any existing damage to the intestinal epithelium, improving barrier function and reducing FITC-d leakage.

The fact that HA-fed poults showed lower FITC-d levels than the positive control further strengthens the evidence for HA’ protective effects [19]. This indicates that HA actively contribute to maintaining gut barrier integrity and reducing permeability, even in the presence of AFB_1_. Moreover, reduced intestinal permeability is crucial for poultry health, as it prevents the entry of harmful pathogens and toxins into the bloodstream [56].

On the other hand, cutaneous basophil hypersensitivity response is a type of delayed-type hypersensitivity reaction mediated by T lymphocytes and basophils. It serves as an indicator of cell-mediated immunity, which is crucial for combating infections and certain types of tumors [57]. In this context, an increase in CBH response in HA-fed poults suggests a possible enhancement of their cell-mediated immunity, despite AFB_1_ exposure. There are other processes that could potentially account for the reported rise in CBH. Previous *in vitro* studies have shown that HA have probiotic properties [53]. In the present study, the prebiotic effect of HA increased the growth of beneficial bacteria from phylum Firmicutes, which can stimulate the immune system and potentially enhance the CBH response. Furthermore, HA possess immunomodulatory properties and can directly activate T lymphocytes and basophils [13], leading to a heightened CBH response. HA binds AFB_1_ via hydrogen bonding, reducing its absorption and potentially mitigating its immunosuppressive effects, allowing the immune system to function more effectively [53]. Indirect effects through the gut barrier were confirmed in the present study by a significant reduction in the serum FITC-d. A healthy gut barrier, potentially supported by HA, can limit the entry of pathogens and toxins, reducing the overall burden on the immune system and allowing it to respond more vigorously to other stimuli, like CBH challenge.

Increased CBH response in HA-fed poults could offer several benefits such as enhanced resistance to infections. A stronger cell-mediated immune system can better fight off bacterial, viral, and parasitic infections, potentially improving overall poultry health and improved vaccine responses. In addition, HA might counteract AFB_1_’s immunosuppressive effects, allowing the immune system to function more effectively, even in hazardous environments.

## 4. Conclusions

In conclusion, this study investigated the impact of dietary HA on intestinal microbiota, gut integrity, ileum morphometry, and immunity in turkey poults fed an AFB_1_-contaminated diet. Our findings clearly revealed that HA supplementation significantly improved intestinal morphology, reduced gut permeability, and modulated intestinal microbiota composition in the AFB_1_-exposed poults. By elucidating the interplay between HA, the gut microbiota, and AFB_1_ in turkey poults, our research holds the potential to develop novel HA-based interventions, leading to more targeted and effective strategies for mitigating aflatoxicosis in poultry production systems, promoting gut health and sustainability by fostering a balanced and resilient gut microbiota and contributing to the overall wellbeing and productivity of turkey poults. Furthermore, HA treatment also enhanced cellular immune response, as evidenced by an increased CBH response. These results suggest that HA have the potential to be used in nutritional interventions to mitigate the detrimental effects of AFB_1_ on poultry gut health and immunity. However, further research is needed to investigate the mechanisms underlying these beneficial effects and optimize the appropriate dose and formulation of HA for practical applications in the poultry industry. Research in this direction is in progress in our laboratories.

## 5. Materials and Methods

### 5.1. Animal Source, Diets, and Experimental Design

A total of 350 1-day-old female Nicholas-700 turkey poults (Aviagen Inc., Fayetteville, AR, USA) were raised in pens for 28 days. Poults were collectively weighted (10 birds/pen), and randomly allocated to one of the five experimental treatments: negative control (basal diet), positive control (basal diet + 250 ng AFB_1_/g), HA (basal diet + 0.25% HA), HA + AFB_1_ (basal diet + HA + 250 ng AFB_1_/g), and Zeolite (ZEO) + AFB_1_ (basal diet + 0.25% Zeolite + 250 ng AFB_1_/g). Each treatment had 7 replicates of 10 poults (*n* = 70). Briefly, a maize–soybean-based turkey poult diet was formulated. AFB_1_, HA, and Zeolite were added to the diet and mixed thoroughly to the specified level. AFB_1_ was produced through the fermentation of rice using an *Aspergillus flavus* strain. The extraction/isolation of HA from vermicompost was done with an alkali solution. A non-commercial zeolitic material was employed as a reference. Details are fully described in the previous study [13]. Poults had *ad libitum* access to water and feed during the experiment. At 28 days, the study was terminated and three poults per replicate (*n* = 21/treatment) were selected to evaluate gut integrity and cellular immunity as described below. One poult per replicate was randomly selected and euthanized by CO_2_ inhalation. Ileum samples were collected to evaluate morphometry and ceca content was collected to evaluate microbiota (*n* = 7/treatment). All animal handling procedures complied with the Institutional Animal Care and Use Committee (IACUC) at the University of Arkansas, Fayetteville (protocol No. 22020).

### 5.2. DNA Extraction and 16S rRNA Gene Sequencing

Microbial DNA in the ceca contents was extracted using a Quick-DNA Fecal/Soil Microbe Miniprep Kit (Zymo Research, Irvine, CA, USA) according to the manufacturer’s instructions. DNA concentration and quality were measured using the NanoDrop ND-1000 (Wilmington, DE, USA). Briefly, the V3–V4 region of the bacterial 16S rRNA gene was amplified using the primers 341F: CCTAYGGGRBGCASCAG and 806R: GGACTACNNGGGTATCTAAT. A library was prepared using NEBNext^®^ Ultra™ Library Prep Kit (New England Biolabs, Ipswich, MA, USA) and subjected to PE250 sequencing on an Illumina HiSeq platform.

### 5.3. Bioinformatics and Statistical Analysis

Raw DNA sequencing reads were analyzed using the QIIME 2 pipeline (v. 2023.07). Briefly, adaptor and primer sequences were removed from each read using the cut–adapt plugin. Paired-end reads were then merged using VSEARCH join pairs and low-quality reads were removed using the quality filter. Sequences were then trimmed to 403 nucleotides and denoised by Deblur [58]. The resulting amplicon sequence variants (ASVs) were then classified into bacterial taxonomy using the Ribosomal Database Project (RDP) 16S rRNA training set (v. 18) and Bayesian classifier. A bootstrap confidence of 80% was used for classification. ASVs with a classification of <80% were assigned the name of the last confidently assigned level followed by “unclassified”. ASV appearing in <5% of samples were removed from the analysis. The top 50 ASVs and all differentially enriched bacteria were further confirmed and reclassified, if necessary, using the EzBioCloud 16S database (v. 2023.08.23. https://www.ezbiocloud.net/identify. accessed on 28 November 2023).

### 5.4. Ileum Morphometry

Ileum samples taken from Meckel’s diverticulum to the ileocecal junction were routinely embedded in paraffin, cut into 5 μm thick sections, and processed using the hematoxylin and eosin (H&E) staining technique. Photomicrographs were acquired using a ICC50W camera associated with a microscope DM2500 Leica (Leica, Wetzlar, Germany). The variables measured were villus height (measured from the top of the villus to the upper part of the lamina propria), villus width (taken at the central part of the villus), and villus area (villus height × villus width). The ImageJ 1.52v software was used for morphometric measurements. In each treatment, 60 measurements were taken per variable.

### 5.5. Gut Integrity

The serum levels of the marker fluorescein isothiocyanate dextran (FITC-d; 3–5 kDa, Merck KGaA, Darmstadt, Germany) were utilized as an indicator of intestinal permeability. For this purpose, twenty-one poults per treatment received a single oral gavage dose of FITC-d (8.32 mg/kg) one hour before they were euthanized. The serum samples were processed according to the recommendations of Baxter et al. [51]. Fluorescence measurements were performed at 485 nm excitation and 528 nm emission using a Synergy HT, multimode micro plate reader (Bio Tek Instruments, Inc., Winooski, VT, USA).

### 5.6. Cellular Immunity

Cutaneous basophil hypersensitivity (CBH) was employed to assess cellular immune activity through the skin response to phytohemagglutinin-M (PHA-M). On day 28, three poults per replicate were randomly selected and injected intradermally in the interdigital skin between the third and fourth digits of the left foot with 0.1 mL of PHA-M (Gibco, Grand Island, NY, USA). The CBH response was calculated by using the following mathematical expression:

$$\begin{array}{c} CBH\left(mm\right)=\left(thickness\;24\;h\;post-injection\right)\\ -\left(thickness\;pre-injection\right) \end{array}$$

### 5.7. Statistical Analysis

The ASV tables were normalized using cumulative sum scaling (CSS) in the metagenomeSeq package of R (v. 1.4.0) [59]. The α-diversity (Shannon’s Index, Observed ASV, and Pielou’s Evenness) and β-diversity (unweighted and weighted UniFrac distances) were calculated using the phyloseq package (v. 1.42.0) [60] and visualized using the ggplot2 package in R. Statistical significance of α-diversity. The relative abundances were determined using a nonparametric Kruskal–Wallis test followed by pairwise Wilcoxon rank-sum test. Significance in β-diversity was calculated using the nonparametric permutational multivariate analysis of variance (PERMANOVA) with the adonis function in the vegan package (v. 2.6.4) [61]. Linear discriminant analysis (LDA) effect size (LEfSe) was performed to identify the differential enrichment of ASVs bacteria among different treatments by using *p* < 0.05 and a LDA score of ≥3.0 as the threshold [62].

Finally, data from the morphometric analysis, serum levels of FITC-d, and the CBH analysis were subjected to ANOVA as a complete randomized design, using the general linear model procedure of SAS [63]. Significant differences among means were determined by using the Tukey multiple range tests at *p* < 0.05.

## Figures and Tables

**Table 1:** Relative abundances (%) of the cecal bacterial phyla and families among different treatments.

Taxon	NC	PC	HA	HA + AFB_1_	ZEO + AFB_1_	SEM *	*p*-Value
**Phyla**							
Firmicutes	79.40 ^b^	74.08 ^b^	86.28 ^a^	88.5 ^a^	80.46 ^b^	2.57	0.007
Proteobacteria	14.11 ^a^	20.71 ^a^	8.79 ^b^	6.92 ^b^	15.02 ^a^	2.44	0.002
Tenericutes	3.85	2.84	1.97	2.71	2.35	0.32	0.696
Cyanobacteria	0.78	1.45	1.38	0.42	0.14	0.26	0.305
Actinobacteria	0.45	0.08	0.23	0.45	0.96	0.15	0.464
**Families**							
Oscillospiraceae	32.75	32.43	32.83	35.87	35.37	0.82	0.836
Lachnospiraceae	32.22 ^ab^	25.76 ^b^	40.28 ^a^	27.42 ^ab^	32.16 ^ab^	2.82	0.049
Enterobacteriaceae	14.11^a^	20.71 ^a^	8.79 ^b^	6.92 ^b^	15.02 ^a^	2.73	0.002
Clostridiales_unidentified	4.90	6.01	4.04	5.05	4.00	0.41	0.261
Erysipelotrichaceae	3.77	1.74	2.06	6.86	3.31	1.02	0.106
Lactobacillaceae	1.66	1.83	2.25	4.44	1.30	0.62	0.283
Mollicutes_unidentified	3.04	2.56	1.63	2.49	1.43	0.34	0.572
Bacillaceae	1.55	1.47	0.16	2.19	1.25	0.37	0.209
Christensenellaceae	0.76	0.80	1.35	2.65	0.83	0.40	0.492
Peptostreptococcaceae	0.41 ^a^	0.29 ^b^	0.61 ^a^	2.32 ^a^	0.71 ^a^	0.41	0.020
Vampirovibrio_unidentified	0.78	1.45	1.38	0.42	0.14	0.28	0.305
Streptococcaceae	0.09	0.97	0.46	0.39	0.11	0.18	0.181
Clostridia_unidentified	0.14	0.85	0.50	0.08	0.15	0.16	0.929
Clostridiaceae 1	0.25	0.29	0.29	0.39	0.42	0.04	0.737
Enterococcaceae	0.67	0.14	0.09	0.16	0.44	0.12	0.072

The mean relative abundances of (%) of top 5 phyla and top 15 families of cecal microbiota of different treatments are shown as means (*n* = 7/treatment). ^a,b^ Indicates significant differences between the treatments within the rows (*p* < 0.05). Statistical significance was determined using the Kruskal–Wallis test, followed by the pairwise Wilcoxon rank sum test. NC—negative control; PC—positive control (basal diet + 250 ng AFB_1_/g); HA—humic acids (basal diet + 0.25% HA); HA + AFB_1_(basal diet + 0.25% HA + 250 ng AFB_1_/g); ZEO—Zeolite (basal diet + 0.25% ZEO + 250 ng AFB_1_/g). * Standard error of the mean.

**Table 2:** Relative abundances (%) of the cecal bacterial genera and ASVs among different treatments.

Taxon	NC	PC	HA	HA + AFB_1_	ZEO + AFB_1_	SEM *	*p*-Value
**Genera**							
*Escherichia/Shigella*	13.16 ^a^	20.68 ^a^	8.70 ^b^	6.92 ^b^	15.01 ^a^	2.43	0.004
*Mediterraneibacter*	10.54	7.49	19.07	10.4	13.27	1.96	0.296
Oscillospiraceae_unidentified	10.23	11.05	11.44	10.69	10.83	0.20	0.943
Lachnospiraceae_unidentified	6.24	6.18	7.47	5.44	6.90	0.35	0.285
*Subdoligranulum*	8.69	4.92	2.44	7.32	4.34	1.11	0.205
*Pseudoflavonifractor*	5.04	5.91	4.39	4.99	4.44	0.27	0.413
Clostridiales_unidentified	4.90	6.01	4.04	5.05	4.00	0.37	0.261
*Enterocloster*	4.64	3.53	5.37	4.16	4.31	0.30	0.693
*Faecalibacterium*	0.17	0.26	4.71	3.74	5.04	1.07	0.848
*Blautia*	4.25	2.67	1.80	1.65	1.83	0.49	0.276
*Anaerostipes*	2.51 ^a^	0.75 ^b^	2.01 ^a^	1.71 ^a^	1.94 ^a^	0.29	0.023
*Eisenbergiella*	1.50	1.55	1.93	1.39	1.33	0.11	0.874
*Acutalibacter*	1.12	1.33	2.11	1.17	1.42	0.18	0.087
*Lactobacillus*	0.86	0.94	1.90	2.41	1.04	0.31	0.148
Mollicutes_unidentified	3.04	2.56	1.63	2.49	1.43	0.30	0.572
**ASVs**							
*Escherichia/Shigella*_F1	12.8 ^a^	20.21 ^a^	8.55 ^b^	6.77 ^b^	14.56 ^a^	2.37	0.004
*Mediterraneibacter*_F2	2.65	2.02	9.08	2.36	2.03	1.37	0.851
*Mediterraneibacter*_F3	2.14	3.31	1.35	2.43	4.21	0.49	0.559
*Subdoligranulum*_variabile_F4	5.10	1.88	0.83	2.18	1.34	0.75	0.815
*Enterocloster*_F5	2.65	1.25	3.04	1.94	2.39	0.31	0.165
*Mediterraneibacter*_F10	0.79	0.37	4.94	2.05	0.99	0.83	0.758
*Faecalibacterium*_F7	0.13	0.24	3.02	2.39	3.18	0.67	0.678
*Lactobacillus_crispatus*_F12	0.86	0.93	1.90	2.37	1.03	0.30	0.148
Mollicutes_unidentified_F9	2.77	1.19	1.33	0.30	1.14	0.40	0.167
*Bacillus*_F8	1.55	1.47	0.16	2.19	1.25	0.33	0.209
*Enterocloster*_F11	1.24	1.36	1.13	1.43	1.36	0.05	0.762
*Oscillibacter*_F15	0.92	1.24	1.96	0.43	1.55	0.26	0.077
*Pseudoflavonifractor*_F14	1.10	1.57	1.23	0.76	1.26	0.13	0.376
*Blautia_obeum*_F18	2.93	1.68	0.40	0.33	0.53	0.50	0.240
*Anaerostipes_butyraticus*_F19	1.77 ^a^	0.56 ^b^	0.74 ^ab^	0.75 ^ab^	1.68 ^a^	0.26	0.044
*Pseudoflavonifractor*_F20	1.35	1.14	0.64	1.27	0.95	0.13	0.666
*Subdoligranulum_*F26	0.50	0.07	0.23	3.37	0.97	0.61	0.161
*Acutalibacter*_F22	0.92	0.89	1.18	0.87	0.99	0.06	0.372
*Pseudoflavonifractor_capillosus*_F16	0.93	1.22	0.63	1.24	0.82	0.11	0.227
*Pseudoflavonifractor*_F21	0.82	1.07	0.88	0.94	1.03	0.05	0.925

The mean relative abundances of (%) of top 15 genera and top 20 ASVs of cecal microbiota of different treatments are shown as means (*n* = 7/treatment). ^a,b^ Indicates significant differences between the treatments within the rows (*p* < 0.05). Statistical significance was determined using the Kruskal–Wallis test, followed by the pairwise Wilcoxon rank sum test. NC—negative control; PC—positive control (basal diet + 250 ng AFB_1_/g); HA—humic acids (basal diet + 0.25% HA); HA + AFB_1_(basal diet + 0.25% HA + 250 ng AFB_1_/g); ZEO—Zeolite (basal diet + 0.25% ZEO + 250 ng AFB_1_/g). * Standard error of the mean.

**Table 3:** Effect of HA on ileum morphometric analysis ^§^, serum levels of FITC-d ^¥^, and cutaneous basophil hypersensitivity response (CBH) in turkey poults consuming a maize–soybean-based diet contaminated with 250 ng AFB_1_/g feed for 28 days.

Parameter	NC	PC	HA	HA + AFB_1_	ZEO + AFB_1_	SEM *	*p*-Value
Villi height (μm)	862.01 ^a^	389.65 ^c^	778.48 ^a^	766.14 ^a^	510.06 ^b^	227.71	<0.0001
Villi width (μm)	118.94 ^b^	146.80 ^ab^	116.85 ^b^	157.43 ^a^	125.88 ^b^	60.85	0.02
Total area (μm^2^)	103.24 ^a^	44.16 ^c^	74.07 ^b^	101.39 ^a^	57.12 ^bc^	36.69	<0.0001
FITC-d (ng/mL)	263.3 ^b^	858.2 ^a^	182.7 ^b^	272.1 ^b^	410 ^b^	669.37	0.01
CBH (mm)	0.37 ^c^	0.50 ^bc^	0.68 ^ab^	0.76 ^a^	0.7 ^a^	0.23	0.0003

^a,b,c^ Means with non-matching superscripts within rows indicates significant difference at *p* < 0.05. Significance was calculated using Tukey multiple range test. ^§^ In each treatment, 60 measurements were taken per variable. ^¥^ Seven replicates/group (*n* = 3 poults per replicate. NC—negative control; PC—positive control (basal diet + 250 ng AFB_1_/g); HA—humic acids (basal diet + 0.25% HA); HA + AFB_1_ (basal diet + 0.25% HA + 250 ng AFB_1_/g); ZEO—Zeolite (basal diet + 0.25% ZEO + 250 ng AFB_1_/g). * Standard error of the mean.

## Data Availability

Upon reasonable request, and subject to review, the authors will provide the data that support the findings of this study. Raw sequencing reads of this study were deposited in the NCBI GenBank SRA database under the accession number PRJNA1020155.

## Supplementary Materials

The following supporting information can be downloaded at: https://www.mdpi.com/article/10.3390/toxins16030122/s1, Figure S1. Effect of humic acids on intestinal morphometric analysis in turkeys consuming a maize–soybean-based diet contaminated with 250 ng AFB/g feed for 28 days. Histological images were taken using a 2.4 × objective on H&E-stained tissue sections. (A) Negative control; (B) positive control; (C) HA; (D) HA + AFB_1_; (E) ZEO + AFB_1_. The black bar shows a representative villi average.
