# Supplementary material for: Assessment of the Impact of Humic Acids on Intestinal Microbiota, Gut Integrity, Ileum Morphometry, and Cellular Immunity of Turkey Poults Fed an Aflatoxin B1-Contaminated Diet
Source: Toxins (Basel). 2024 Feb 29;16(3):122. doi: 10.3390/toxins16030122 (PMC10975313; doi:10.3390/toxins16030122)
Supplement: Supplementary file 1 [file toxins-16-00122-s001.zip › toxins-2868564-supplementary.pdf]

toxins

Assessment of the Impact of Humic Acids on Intestinal Microbiota, Gut Integrity, Ileum Morphometry, and Cellular Immunity of Turkey Poults Fed an Aflatoxin B_1-Contaminated Diet

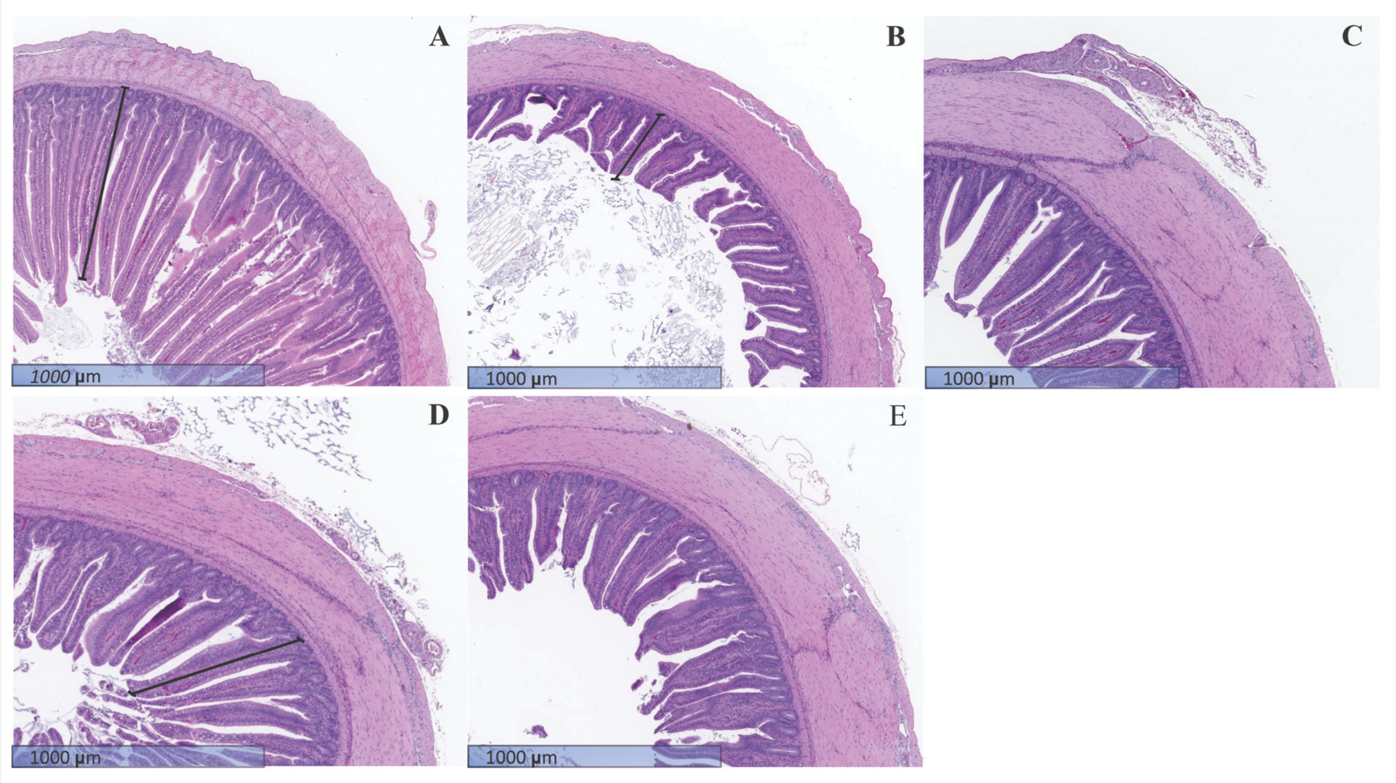

Figure S1. Effect of humic acids on intestinal morphometric analysis in turkeys consuming a maize–soybean-based diet contaminated with 250 ng AFB/g feed for 28 days. Histological images were taken using a 2.4 × objective on H&E-stained tissue sections. (A) negative control; (B) positive control; (C) HA; (D) HA + AFB_1; (E) ZEO + AFB_1. The black bar shows a representative villi average.
